# PnLRR-RLK27, a novel leucine-rich repeats receptor-like protein kinase from the Antarctic moss *Pohlia nutans*, positively regulates salinity and oxidation-stress tolerance

**DOI:** 10.1371/journal.pone.0172869

**Published:** 2017-02-27

**Authors:** Jing Wang, Shenghao Liu, Chengcheng Li, Tailin Wang, Pengying Zhang, Kaoshan Chen

**Affiliations:** 1 School of Life Science and National Glycoengineering Research Center, Shandong University, Jinan, China; 2 Marine Ecology Research Center, The First Institute of Oceanography, State Oceanic Administration, Qingdao, China; 3 Shandong Provincial Key Laboratory of Carbohydrate Chemistry and Glycobiology, Jinan, China; National Taiwan University, TAIWAN

## Abstract

Leucine-rich repeats receptor-like kinases (LRR-RLKs) play important roles in plant growth and development as well as stress responses. Here, 56 LRR-RLK genes were identified in the Antarctic moss *Pohlia nutans* transcriptome, which were further classified into 11 subgroups based on their extracellular domain. Of them, PnLRR-RLK27 belongs to the LRR II subgroup and its expression was significantly induced by abiotic stresses. Subcellular localization analysis showed that PnLRR-RLK27 was a plasma membrane protein. The overexpression of PnLRR-RLK27 in *Physcomitrella* significantly enhanced the salinity and ABA tolerance in their gametophyte growth. Similarly, PnLRR-RLK27 heterologous expression in *Arabidopsis* increased the salinity and ABA tolerance in their seed germination and early root growth as well as the tolerance to oxidative stress. PnLRR-RLK27 overproduction in these transgenic plants increased the expression of salt stress/ABA-related genes. Furthermore, PnLRR-RLK27 increased the activities of reactive oxygen species (ROS) scavengers and reduced the levels of malondialdehyde (MDA) and ROS. Taken together, these results suggested that PnLRR-RLK27 as a signaling regulator confer abiotic stress response associated with the regulation of the stress- and ABA-mediated signaling network.

## Introduction

Land plants are constantly challenged by environmental stresses such as drought, salinity and extreme temperature, which can cause irreversible damage to plants intracellular structures by severe dehydration [1]. Plant internal alterations in response to environmental signals mostly depend on a sophisticated signaling network. Membrane anchored receptor-like kinases (RLKs) are the key regulators to active such signaling pathways by perceiving and processing external stimuli to cellular signaling molecules [2]. So far, 610 RLKs in *Arabidopsis* and 1100 RLKs in rice were identified, making up over 2% of each genome, and the significant expansion of this family has been believed to be crucial for plant-specific adaptations [3–4]. However, only a few RLKs have been identified to play roles in plant growth and development, pathogens defense and abiotic stress [2,5]. Knowledge about RLKs-mediated signal transduction may lead to continued development of rational breeding and transgenic strategies to improve stress tolerance [4].

The leucine-rich repeats protein kinases (LRR-RLKs), which are the largest subgroup of the RLK family with more than 235 members in *Arabidopsis* and 309 members in rice, contain the N-terminal leucine-rich repeats domain, a single transmembrane domain and a C-terminal kinase domain [3,5–8]. The LRR-RLKs are the primary regulatory in perception and processing of extracellular stimuli finally leading to the expression of the stress-responsive target genes to generate the adaptive molecular mechanism [9]. Generally, they perceive extracellular signals through the LRR domain and transmitted the signals via their Ser/Thr kinase domains [10]. The data collected so far indicates that LRR-RLKs from monocots and dicotyledons participated in diverse signaling processes, including plant meristems size regulation, organ growth, pathogen defense and hormone perception [11–18]. In addition, the LRR-RLKs have also been found to play important roles in regulating plants responses to abiotic stress. Several LRR-RLKs involved in plants abiotic stress responses have been identified in the molecular levels. The *Medicago truncatula Srlk* was identified to improve plants roots salt stress tolerance by accumulating fewer sodium ions and reducing the expression level of several salt-responsive genes [19]. The OsSIK1 transgenic rice overexpression plants showed higher tolerance to salt and drought stresses by activating the antioxidative system, and displayed less stomatal density in leaves [20]. The GsLRPK possessed kinase activity in the presence of cold stress and enhanced the resistance to cold stress by increasing the expression of cold responsive genes [21]. Furthermore, the latest identified LP2 functioned as a negative regulator of drought stress by directly regulating the drought-related transcription factor DST and interacting with the drought-responsive aquaporin proteins, while overexpressing LP2 in rice reduced the H_2_O_2_ levels and inhibited the stomatal closure in leaves [22].

Mosses, the dominate Antarctic vegetation, are found in ice-free areas where sufficient summer snowmelt occurs [23]. To survive and adapt to the extreme climates, mosses have established a variety of adaptive strategies to protect them from various stresses. For example, people found that the Antarctic mosses have some fascinating abilities to well adapt to high light stress and low environmental temperatures by protection of its photosystems using the xanthophyll cycle [24]. The soluble carbohydrates in the Antarctic mosses function as osmoprotectors in response to water stress; the content of the non-structural carbohydrates or the raffinose family oligosaccarides decreased during desiccation and increased during rehydration [25]. Furthermore, cell wall-bound insoluble phenylpropanoids as a more passive UV-screening mechanism also will increase the tolerance of Antarctic mosses to high ultraviolet radiation [26]. In addition, the Antarctic mosses usually produces more secondary metabolites such as UV-B absorbing flavonoids and carotenoids, which act as antioxidants and stimulator of DNA repair processes, to protect their biological systems against UV radiation [27]. However, the signaling networks that how the Antarctic mosses sense the extreme environment and transfer signals to intracellular signaling molecules are still unclear.

In this study, we isolate a LRR-RLK gene (*PnLRR-RLK27*) from the Antarctic moss *Pohlia nutans*. PnLRR-RLK27 overexpression in bryophyte *physcomitrella patens* and heterologous expression in *Arabidopsis* enhanced the tolerance to salt stress. This effect appears to operate through the regulation of ROS and ABA signaling pathways.

## Materials and methods

### Plant materials, growth conditions and stress treatments

The Antarctic moss *Pohlia nutans* was collected from the terrane near the Great Wall Station on King George Island (S62°13.260′, W58°57.291′). Accoding to The Antarctic Treaty, no specific permissions were required for collecting samples on this location. The Antarctic moss samples (a few shoots and roots with soil matrix) were placed in vacuum-sealed plastic bags, stored at 4°C for transportation. Our field studies (i.e. moss sample collection) did not involve any endangered or protected species. The mosses were then cultured in the pots soil with the mixture of Base Substrate (Klasmann-Deilmann, Geeste, Germany) and local soil (1:1) in a greenhouse at 16°C, 70 μmol·m^-2^·s^-1^ light and 70% relative humidity. The aerial portions of mosses cultivated under this condition were used as control. For cold stress, mosses were treated at 4°C for the indicated times. For salt, dehydration and abscisic acid (ABA) stress, mosses were sprayed with 200 mM NaCl, 20% (w/v) polyethylene glycol 6000 solution (PEG) or 50 μM ABA for the indicated times, respectively.

*Arabidopsis thaliana* (ecotype Columbia, Col-0) plants used for transformation were grown in the chamber at 22°C under 16 h light, light intensity of 80 μE·m^-2^·s^-1^, and 60% relative humidity in the growth phase. After the growth of 4 weeks with 8 h light, they were transfered to the long light greenhouse with 16 h light for the plants transformation and maturation.

*Physcomitrella patens* were grown on BCD medium at 25°C with 16 h light [28]. After 2 weeks, gametophyte tissues were uniformly cut by a tissue homogenizer and then evenly distributed on cellophane overlaid BCD medium supplemental with 5 mM diammonium (+) tartrate to form protonema. The new protonema was used for the plants transformation.

### Isolation and bioinformatics characterization of PnLRR-RLK27 gene

Previously, we performed the Antarctic moss *P*.*nutans* tanscriptome sequencing by the Illumina Hiseq 2500 platform and analyzed the gene expression profile of *P*.*nutans* after salt stress (data not shown). To fetch LRR-RLK proteins, the HMMER program was used to search the *P*. *nutans* transcriptome [29]. All of the retrieved LRR-RLK proteins were then subjected to BLAST and SMART databases for annotation of the domain structure. Only candidate (Hmmsearch E-value<1.0 e^-10^) containing at least a LRR domain and one protein kinase domain was identified as a “true” LRR-RLK.

The protein structure was predicted by the SMART web site (http://smart.embl-heidelberg.de/) [30]. Homology searches were subjected to BLAST (http://www.ncbi.nlm.nih.gov/blast/) against the non-redundant protein databases (nr). Multiple alignments were generated with the Clustal W program. The phylogenetic trees were performed using the Mega 4.0 software with the neighbor-joining method and 1000 bootstrap replicates [31].

### RNA extraction and real-time quantitative PCR analysis

Total RNA was extracted from moss gametophyte tissues or *Arabidopsis* seedlings using CTAB method and Trizol reagent (Takara), respectively [32]. First-strand cDNA was synthesized using 5 μg of total RNA by Easy Script^™^ RT-PCR kit (abm, Canada). Real-time PCR reactions were performed using the Bestar^®^ SybrGreen qPCR mastermix (DBI Bioscience). The cycling regime was composed of a denaturation step of 94°C/2 min, followed by 40 cycles of 94°C/20 s, 56°C/30 s, 72°C/20 s. A melting-curve analysis was performed over the range 65°C to 95°C at 0.5°C intervals. Relative gene expression levels were calculated using the comparative Ct (2^−ΔΔCt^) method [33]. The primers used in Real-time PCR are presented in S1 Table.

### Subcellular localization analysis of the PnLRR-RLK27 protein in *Physcomitrella patens* and *Arabidopsis*

The full-length sequence of *PnLRR-RLK27* without the stop codon was amplified with the pair primers (S1 Table). Then the *pBI221*:*p35S*:*PnLRR-RLK27*:*GFP* or/and *pBI221*:*p35S*: *H*^*+*^*-ATPase*:*RFP* was constructed and transfected into the protoplasts isolated from 5 day-old wild-type *P*. *patens* protonema or 4 week-old wild-type *Arabidopsis* Col-0, respectively [34–35]. Meanwhile, the *pBI221*:*p35S*:*GFP* or/and *pBI221*:*p35S*:*H*^*+*^*-ATPase*:*RFP* vector was transformed as a control. The transformed *P*. *patens* and *Arabidopsis* protoplasts were incubated in the dark at 22°C for 2 d and 10 h, respectively. GFP signal was observed with a confocal laser scanning microscopy (LSM700, Carl Zeiss, Shandong University) at excitation wavelengths of 488 nm. Chlorophyll autofluorescence and RFP signal were obtained with an excitation at 647 nm and 543 nm, respectively.

### Generation of PnLRR-RLK27 transgenic *Physcomitrella patens* and transgenic *Arabidopsis* plants

To generate the transgenic forms of *P*. *patens*, the *PnLRR-RLK27* sequence was inserted into the *pTFH15*.*3* vector (obtained from Drs.Tomoaki Nishiyama and Mitsuyasu Hasebe) according to the method described by Yin [36]. The protoplasts were isolated according to the protocol of Cove with some modification [28]. Polyethylene glycol (PEG)-mediated DNA uptake was used for transformation of the moss (*P*. *patens*) protoplasts with some key modification [37]. Briefly, 30 μg of linearized *pTFH15*.*3*:*PnLRR-RLK27* homologous recombinant vector were mixed with 300 μL protoplast solution and 300 μL PEG solution (40% PEG 4000 and 0.1 M Ca(NO_3_)_2_·4H_2_O in 3M medium, pH 5.6). The mixture was heated in a water bath at 45°C for 5 min and immediately transfered to metal bath at 20°C with gentle shaking for 5 min. After gradient diluted with 8% D-mannitol solution, the transformed mixture was dispensed to the cellophane covered PRMB agar medium. After incubated at 25°C for 7 d in the 16 h light, the cultures were transferred to selection BCD solid medium containing 25 μg·mL^-1^ G418 (Sigma) for 2 weeks, followed by a release period of 10 d on standard medium and two addtional selection period with 50 μg·mL^-1^ or 100 μg·mL^-1^ G418 for 2 weeks. Plants surviving the third round of selection were counted as stable transformants, and confirmed by RT-PCR analysis using specific gene primers (S1 Table).

To construct the transgenic *PnLRR-RLK27 Arabidopsis* plants, the full-length *PnLRR-RLK27* sequence was ligated into the XbaI and KpnI cloning sites of the modified binary *pROK2* vector. The vector was introduced into the *Agrobacterium tumefaciens* strain EHA105, and then transformed into *Arabidopsis* Col-0 plants by floral-dip method [38]. Transformants were selected based on their resistance to kanamycin. Homozygous T3 or T4 transgenic seedlings were used for phenotype and gene expression analysis.

### Abiotic stress tolerance analysis of PnLRR-RLK27 transgenic *Physcomitrella patens* and *Arabidopsis* plants

For phenotype analysis of transgenic *P*. *paten*, gametophytes of three *PnLRR-RLK27* transgenic plants and wild type plants were growth for 30 d in a climate chamber at 25°C. Then the same size stem tip were cut and placed on BCD solid medium containing different concentrations of NaCl or ABA. After cultured 4 weeks at 25°C with 16 h light, the plants size was measured and the visual phenotypes were photographed. The experiments were replicated three times.

For phenotype analysis of transgenic *Arabidopsis*, seeds surface-sterilized with 70% (v/v) ethanol for 5 min and 95% (v/v) ethanol for 25 s were plated on 1/2 MS solid medium supplemented with different concentrations of NaCl, ABA and H_2_O_2_, kept in the dark at 4°C for 2 d to break dormancy, and subsequently transferred to a 16 h photoperiod at 22°C. The seedlings roots length was measured. A germination assay was conducted by plating surface-sterilized seeds on 1/2 MS solid medium containing various concentrations of NaCl and ABA. After stratification at 4°C in dark for 2 d, the seeds were incubated at 22°C under light to allow germination. The emergence of an open green cotyledons was taken as representing a successfully germination seeds. Germination rates were expressed as the proportion of seeds that had successfully germinated. The experiments were replicated three times.

### Stomatal aperture assays of PnLRR-RLK27 transgenic *Arabidopsis*

The rosette leaves of 3 week-old *Arabidopsis* plants were floated in the solution containing 10 mM KCl, 50 μM CaCl_2,_ 10 mM MES, pH 6.15, and exposed to light for 2.5 h to make the stomata completely open. Subsequently, 0 or 50 μM ABA was added to the solution for stomatal closing. After ABA treatment for 2.5 h, the epidermal strips were peeled from the rosette leaves using a thin-tipped forceps and fixed by coverslip. Stomatal apertures in epidermal peels were photographed by the microscope and the width of stomatal apertures was measured with the Image J [39–40]. Each sample was replicated three times.

### Measurement of H_2_O_2_, MDA, proline content and enzyme SOD and POD activities

H_2_O_2_ level was detected via DAB staining as described previously [41]. The leaves of 4 week-old wild type and transgenic plants were irrigated with 200 mM NaCl solution for 24 h. Then the leaves of them were isolated and immersed in 1 mg·mL^-1^ DAB with 10 mM sodium phosphate buffer solution (pH 3.8) at 22°C in dark until appearing a clearly reddish brown spots. Finally, the leaves were bleached and the brown spots were fixed and visualized with 95% ethanol solution by boiling bath. The brown spots were characteristic of DAB reacting with H_2_O_2_.

In addition, SOD and POD activities were assayed according to the method as described in references [42–43]. Briefly, plants were treated with 200 mM NaCl for 24 h and 0.5 g of the same layered leaves were ground in an ice-cold mortar using 50 mM potassium phosphate buffer (pH 7.4). After 13 000 g centrifugation at 4°C for 20 min, the supernatant was used for detecting SOD and POD activities.

The content of MDA (malondialdehyde) was tested for indirect calculating lipid peroxidation using thiobarbituric acid method described by Hodges [44]. 0.5 g of seedlings were ground and extracted with 5 mL of 20% (w/v) trichloroacetic acid and centrifuged at 10 000 g for 10 min. 1 mL of the supernatant was added with 2 mL of thiobarbituric acid solution (0.5% (w/v) in 20% TCA). The mixture was incubated at 100°C for 10 min, then cooled in ice and centrifuged at 10 000 *g* for 15 min. The absorbance of plants supernatant extract was measured at 450, 532 and 600 nm wavelength. The concentration of MDA was calculated using the following equation: concentration (μmol·L^-1^) = 6.45 × (OD_532_ − OD_600_) 0.56 × OD_450,_ where OD represents optical density. The experiment was repeated three times.

Proline contents were measured as described in Bates et al [45]. Briefly, 0.5 g of 2 week-old seedlings were weighed and extracted in 5 mL of 3% aqueous sulfosalicylic acid at 100°C for 10 min. After 10 000 g centrifugation for 10 min, a 1:1:1:2 (v/v/v/v) solution of plant extract, distilled water, glacial acetic acid and 2.5% (w/v) acid-ninhydrin was incubated at 100°C for 1 h. The reaction was then cooled at normal temperature and the proline was extracted with 2:1 (v/v) solution of toluene and plant extract. The mixture was vortexed for 20 s to extract red products and their absorbance was measured at 520 nm wavelength. Standard curve of different concentration of proline was prepared using the same method to measure proline content of the samples. The content of proline on a fresh weight was calculated using the following equation: content (μg·g^-1^) = (w_standard proline_ × v _total sample extract_) / (w_sample_ × v _experimental sample extract_). The experiment was repeated three times.

### Statistical analysis

The data of *PnLRR-RLK27* transcriptional levels, seedling root length, seed germination and related genes expression were subjected to Student’s *t* test analysis using a one-way ANOVA. Error was measured by Standard Error of the Mean (SEM) and significant difference was set at P<0.05.

## Results

### Identification of PnLRR-RLK gene family and phylogenetic analysis of PnLRR-RLK27

**Fig 1:**
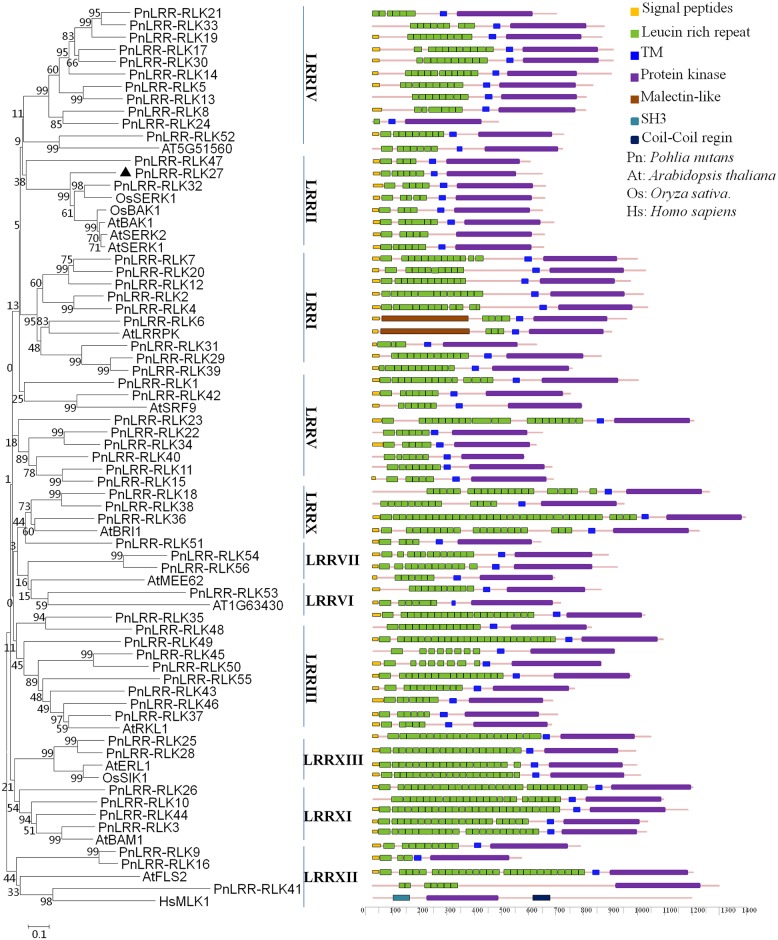
The relationships between PnLRR-RLK protein kinases with other eukaryotic genomes. The kinase domain amino acid sequences of PnLRR-RLKs genes and LRR-RLK kinases family representatives from *Arabidopsis*, *Oryza sativa* and human were used for generating the neighboring-joining tree with 1,000 bootstrap replicates. The PnLRR-RLKs family forms a close relationship to *Arabidopsis*, *Oryza sativa* kinases and *Homo sapiens* receptor Tyr kinase HsMLK1.

A total of 56 putative *LRR-RLK* genes were identified from the Antarctic moss *P*. *nutans* transcriptome (Fig 1). These *LRR-RLK* genes were sequential named from PnLRR-RLK1 to PnLRR-RLK56 by their E-value of the Protein kinase domain (PF00069) which generated by Hmmsearch program (Fig 1). These genes had been submitted to the Genebank, and the accession numbers were listed in S2 Table. According to the differential expression analysis of the Antarctic moss *P*. *nutans* transcriptome, 4 LRR-RLK genes were upregulated and 10 were downregulated after salt treatment (S2 Table). Among them, PnLRR-RLK27, which has the largest 3.18-fold change of transcription level after salt treatment, was selected for further study.

Plant LRR-RLKs belong to a monophyletic gene family that distinct from other families of eukaryotic protein kinases [6]. Kinase domains of RLKs are very conserved, and used to distinguish the subfamilies of the diverse LRR-RLKs [3,7]. To study the evolutionary relationships and obtain the functional data of PnLRR-RLK members, a phylogenetic analysis was conducted using the kinase domains sequence. The phylogenetic tree showed that these PnLRR-RLKs could be classified into 11 major subgroups (LRR I-XIII) according to the *Arabidopsis* homologues (Fig 1). In which, the PnLRR-RLK27 was relatively close to *Arabidopsis* and *Oryza sativa* LRR II family proteins which were reported to be induced by various stresses (Fig 1) [46]. Furthermore, to further understand the potential functions of the PnLRR-RLK genes, all putative motifs of these proteins were investigated using the BLAST and SMART databases. As shown in Fig 1, 45 PnLRR-RLKs had the signal peptides, leucine-rich repeat domain, transmembrane domain and kinase domain, while 11 PnLRR-RLKs (PnLRR-RLK10, 11, 13, 18, 22, 33, 38, 40, 41, 45 and 48) missed signal peptides and PnLRR-RLK41 missed TM domain. PnLRR-RLK27 contained a signal peptide (SP) at the N-terminal portion (amino acids 1–28), 6 tandem copies of 36- or 23-amino acid leucine-rich repeat (amino acids 32–170) in the extracellular domain, a transmembrane domain (TM) (amino acids 233–255) and a serine/threonine protein kinase domain (KD) (amino acids 295–570) with I-XI conserved subdomains in the C-terminal cytoplasmic region (Fig 1) [30,47]. Moreover, the protein kinase domain of PnLRR-RLK27 possessed the conserved ATP binding site and active site suggested that PnLRR-RLK27 might functions as a potential kinase.

### The expression of PnLRR-RLK27 was induced by multiple abiotic stress

**Fig 2:**
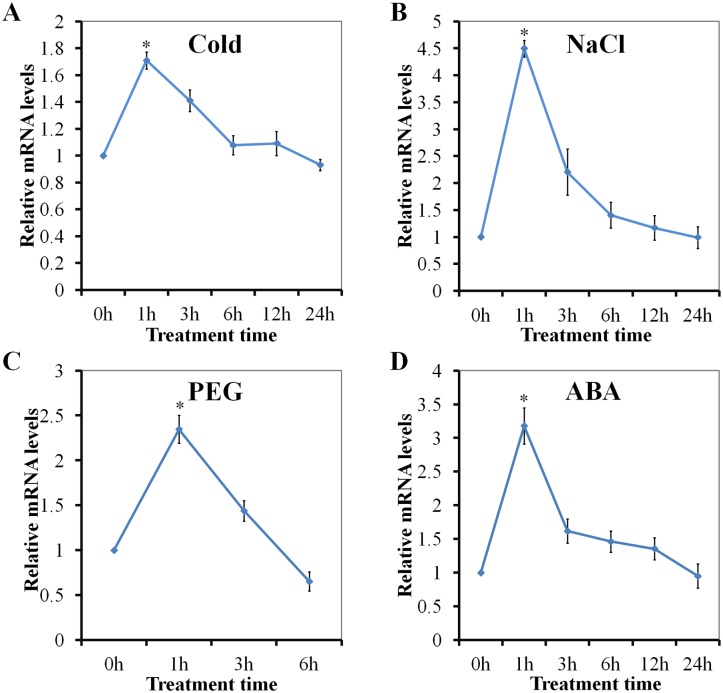
Expression patterns of PnLRR-RLK27 in the Antarctic moss *Pohlia nutans* in response to different stress treatments analyzed by real-time PCR. (A) 200 mM NaCl treatment; (B) Cold treatment (4°C); (C) 20% PEG6000 treatment for simulated drought stress; (D) 50 μM abscisic acid (ABA) treatment. In each stress treatment, the different time point samples were all from the same plant pot cultures. Vertical bars indicate mean ±SE of three replicates of the sample. Asterisks (*) indicate statistically significant differences with the control group at *P*<0.05.

In the Antarctic environment, moss can be exposed to freezing temperatures (below -7°C) while in full sunlight, particularly in the late summer months when the snow cover has melted [48]. Real-time PCR analysis showed that the expression levels of *PnLRR-RLK27* reached maximum value at 1 h, but then declined at 3 h after cold treatment (Fig 2A). Salinity levels can also affect the growth of vegetation, as many species grow near the Antarctic coast. Real-time PCR analysis confirmed that *PnLRR-RLK27* transcript accumulated significantly after 200 mM NaCl treatment for 1 h (Fig 2B). High salinity also can cause osmotic stress [49]. In the presence of 20% polyethylene glycol 6000 (PEG6000) to simulate osmotic stress, the expression levels of *PnLRR-RLK27* were significantly increased at 1 h (Fig 2C). Abiotic stresses could cause the production of plant hormone ABA and then mediate downstream stress-related signaling pathways [50]. The *PnLRR-RLK27* expression were also induced by ABA treatment and it reached the highest levels with the ABA treatment for 1 h, but then showed a significant decrease after 1 h ABA treatment (Fig 2D). These results implied that *PnLRR-RLK27* might involve in salt, ABA, cold, drought and possibly other osmotic stress signaling pathways, and serve as a regulator in the abiotic stress response.

### Multiple sequence alignment and subcellular localization of the PnLRR-RLK27

**Fig 3:**
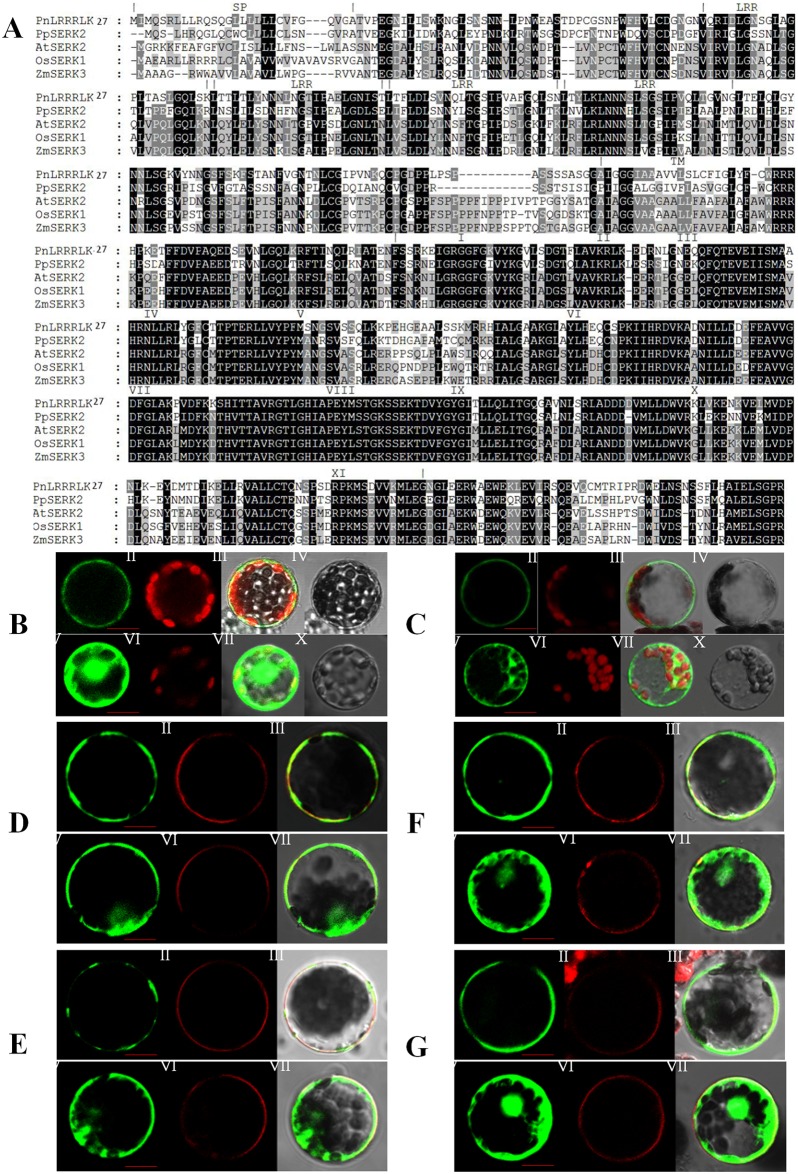
Sequence alignment and subcellular localization of PnLRR-RLK27 in *Physcomitrella patens*. (A) Amino acid sequence alignment between PnLRR-RLK27 and other LRR-RLK family members *Physcomitrella patens* subsp. *patens* (PpSERK2), *Arabidopsis thaliana* (AtSERK2), *Oryza sativa* (OsSERK1), *Zea mays* (ZmSERK3). Multiple sequence alignments were conducted using ClustalW. Black boxes show identical amino acid residues, and gray shades show similar residues. Deletions are indicated by dashes to allow maximum alignment. (B and C) The localization of p35S:PnLRR-RLK27:GFP in *Physcomitrella patens* and *Arabidopsis* protoplasts. (D and F) The localization of p35S:PnLRR-RLK27:GFP in *Physcomitrella patens* and *Arabidopsis* protoplasts in the presence of FM4-64. (E and G) The colocalization of p35S:PnLRR-RLK27:GFP and H^+^-ATPase:RFP in *Physcomitrella patens* and *Arabidopsis* protoplasts. (B to G I and V) Green fluorescence of PnLRR-RLK27-GFP fusion protein or GFP protein; (B to C II and VI) Red autofluorescence of chloroplasts. (D to F II and VI) Red autofluorescence of plasma membrane in the presence of FM4-64. (E to G II and VI) Red autofluorescence of H^+^-ATPase:RFP fusion protein. (B to G III and VII) Merged image of green fluorescence, bright field, and red autofluorescence. (B to C IV and X) The protoplast in bright field. *Bar* 10 μm.

The identity of PnLRR-RLK27 amino acid sequence with other species LRR-RLKs varied from 56.0% to 65.8%. It shared homology with *P*. *patens* subsp. *patens* PpSERK2 (65.8%), *Arabidopsis* AtSERK2 (56.0%), *Oryza sativa* OsSERK1 (57.0%) and *Zea mays* ZmSERK3 (57.0%) (Fig 3A). The subcellular localization of the PnLRR-RLK27 protein was investigated using *P*. *patens* protoplasts and *Arabidopsis* mesophyll protoplasts with constructs expressing the *pBI221*:*p35S*:*PnLRR-RLK27*:*GFP* fusion. In the control *pBI221* vector transformed protoplasts, GFP proteins distributed evenly in the cell membrane, the cytoplasm and nucleus (Fig 3B to 3GV). However, in the *pBI221*:*p35S*: *PnLRR-RLK27*:*GFP* transformed protoplasts, GFP proteins was only found in the plasma membrane (Fig 3B to 3GI). Consistently, the pBI221:p35S:PnLRR-RLK27:GFP proteins colocalized with both the lypophilic dye N-(3-triethylammoniumpropyl)-4-(6-(4-(diethylamino) phenyl) hexatrienyl) pyridinium dibromide (FM4-64) (Fig 3D to 3EI and 3EII) and plasma membrane marker H^+^-ATPase-RFP protein [51] in the plasma membrane (Fig 3F to 3GI and 3GII). These results clearly indicated that the PnLRR-RLK27 protein localized on the plasma membrane.

### Overexpression of the PnLRR-RLK27 in *Physcomitrella patens* and *Arabidopsis* enhanced plant tolerance to salinity

**Fig 4:**
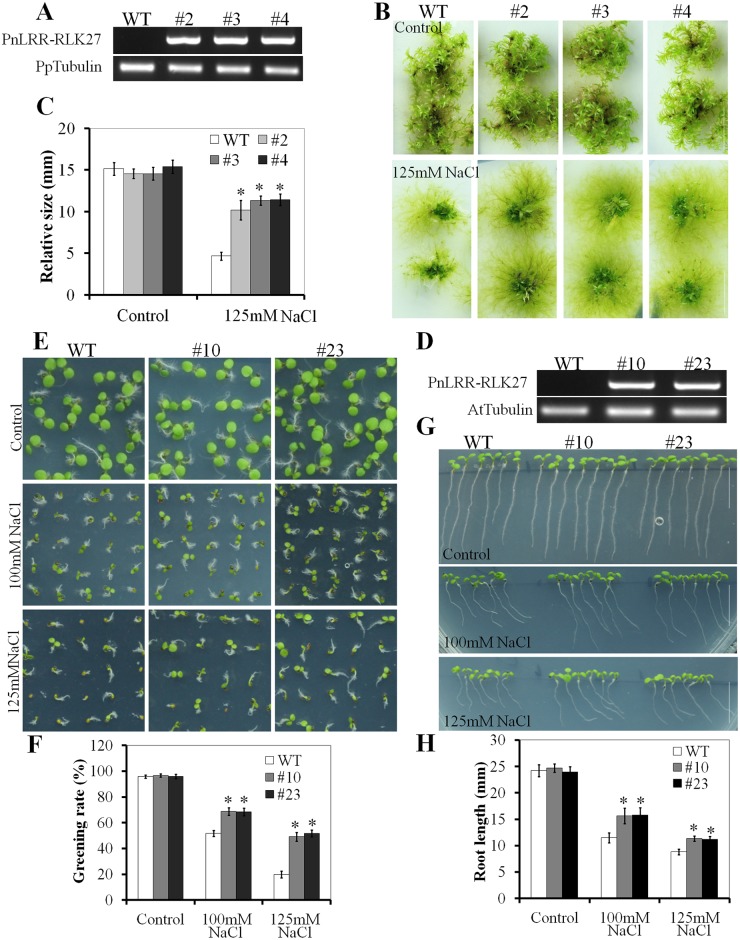
PnLRR-RLK27 contributes to the salt tolerance in transgenic *Physcomitrella patens* and *Arabidopsis*. (A) RT-PCR analysis revealed that the *PnLRR-RLK27* was successfully transcribed in *Physcomitrella patens*. (B) The size of transgenic *Physcomitrella patens* gametophyte plants was significantly larger than that of the wild type under salt stress conditions (4-week-old plants). (C) Statistical analysis of gametophyte size as shown in (B). (D) RT-PCR analysis revealed that the *PnLRR-RLK27* was successfully transcribed in *Arabidopsis*. *Tubulin* gene was used as internal references. (E) The seed germination rate of transgenic seedlings was significantly higher than that of the wild type under salt stress conditions (6 days' seed germination). (F) Statistical analysis of seed germination rates as shown in (E). Seed germination rates were calculated by counting the proportion of the WT plants and the transgenic seedlings bearing open green cotyledons. (G) PnLRR-RLK27 promotes the growth of *Arabidopsis* seedling after salinity treatment. (H) Measurement of root length of salinity-stressed *Arabidopsis* seedlings shown in (G). Vertical bars are presented as means ±SE, and asterisks (*) indicate significant differences of means between the transgenic lines and the WT plants at *P*<0.05. *Bar* 10 mm.

To further elucidate the role of *PnLRR-RLK27* in response to abiotic stress, it was transformed into moss plant *P*. *patens* and into *Arabidopsis* ecotype Columbia (Col-0). Three independent transgenic plants *P*. *patens* (#2, #3 and #4) were identified by RT-PCR using gene specific primers (Fig 4A). The same size stem tips of control and transgenic gametophytes were placed on BCD medium containing 0 or 125 mM NaCl. On standard BCD medium, the transgenic *P*. *patens* plants and the wild-type plants grew equally well, and their seedling phenotypes were indistinguishable (Fig 4B). However, the growth rates of the three transgenic gametophytes were significantly higher than the WT plants on 125 mM NaCl medium. The clone size of three transgenic gametophytes (#2, #3 and #4) were 10.2, 11.1 and 11.4 mm respectively, while the WT plants was 4.7 mm at 7 weeks on 125 mM NaCl medium (Fig 4C).

The *Arabidopsis* transgenic *PnLRR-RLK27* plants (#10 and #23) were identified by RT-PCR using gene specific primers (Fig 4D). There was no obvious phenotypic difference between two transgenic plants (#10 and #23) and the WT plants. However, on 1/2 MS solid medium containing 100 or 125 mM NaCl, the germination rate of two over-expressing lines was significantly higher than the WT plants (Fig 4E). The germination rates of the two transgenic plants (#10 and #23) were 49.1% and 51.6% after 3 d compared to 20.0% of the WT plants at the 125 mM NaCl (Fig 4F). The root length assays showed that the two transgenic plants (#10 and #23) did not have obvious phenotypic difference when compared to the WT plants on 1/2 MS solid medium. However, in the presence of 100 or 125 mM NaCl, the two transgenic plants (#10 and #23) were more tolerant than the WT plants, forming longer primary roots (1.4-fold longer at 100 mM NaCl and 1.3-fold longer at 125 mM NaCl) (Fig 4G and 4H).

### Overexpression of the PnLRR-RLK27 decreased the sensitivity to ABA

**Fig 5:**
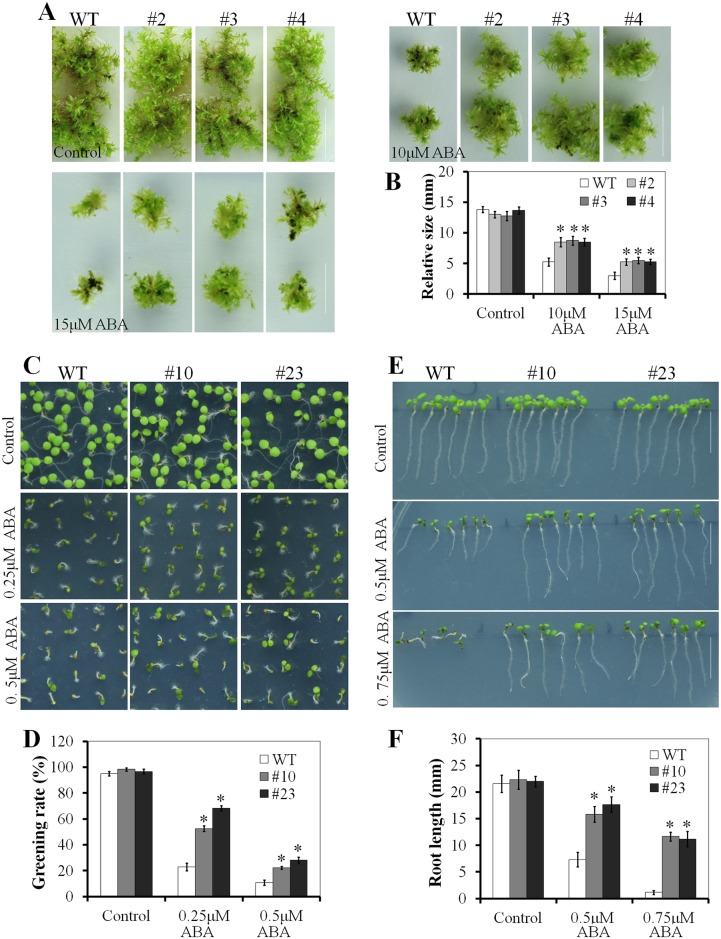
PnLRR-RLK27 reduces the ABA sensitivity in transgenic *Physcomitrella patens* and *Arabidopsis*. (A) The size of transgenic *Physcomitrella patens* gametophyte plants was significantly larger than that of the wild type after ABA treatment (4 week-old plants). (B) Statistical analysis of gametophyte size as shown in (A). (C) Seed germination of transgenic lines were significantly higher than that of the wild type under different concentrations of ABA (4 days' seed germination). (D) Statistical analysis of the transgenic *Arabidopsis* seedling greening shown in (C). (E) PnLRR-RLK27 promotes the growth of *Arabidopsis* seedling after ABA treatment. (F) Statistical analysis of the root length in transgenic *Arabidopsis* shown in (E). Vertical bars are means ±SE, and asterisks (*) indicate significant differences of means between the transgenic lines and the WT plants at *P*<0.05. *Bar* 10 mm.

Salt stress usually induces plant to increase the levels of endogenous ABA [52]. Thereafter, the increased ABA may inhibit seed germination and plant development. As shown in Fig 5A, the transgenic *P*. *patens* plants (#2, #3 and #4) were highly insensitive to the provision of exogenous ABA. The clone size of the transgenic *P*. *patens* plants (#2, #3 and #4) were higher than the control plants when exposed to 10 or 15 μM ABA. At the BCD solid medium containing 10 μM ABA, the gametophyte sizes of the transgenic *P*. *patens* plants was 1.6-fold larger than that of the WT plants at 10 μM ABA, while 1.8-fold larger at 15 μM ABA (Fig 5B). In *Arabidopsis*, root resistance assays showed that two transgenic plants (#10 and #23) were also insensitive to ABA, forming the longer primary roots. The root length of the transgenic plants was 2.2-fold longer than that of the WT plants at 0.5 μM ABA, while 10-fold longer at 0.75 μM ABA (Fig 5C and 5D). The germination rate assays also showed that two transgenic plants (#10 and #23) were insensitive to ABA (Fig 5E). The germination rates of two transgenic plants were 52.5% and 68.3% compared to 22.9% in the WT plants at 0.25 μM ABA, while the germination rates of two transgenic plants were 22.3% and 28.0% compared to 10.8% in the WT plants at 0.5 μM ABA (Fig 5F).

### PnLRR-RLK27 affected the stomatal movement under ABA treatment

**Fig 6:**
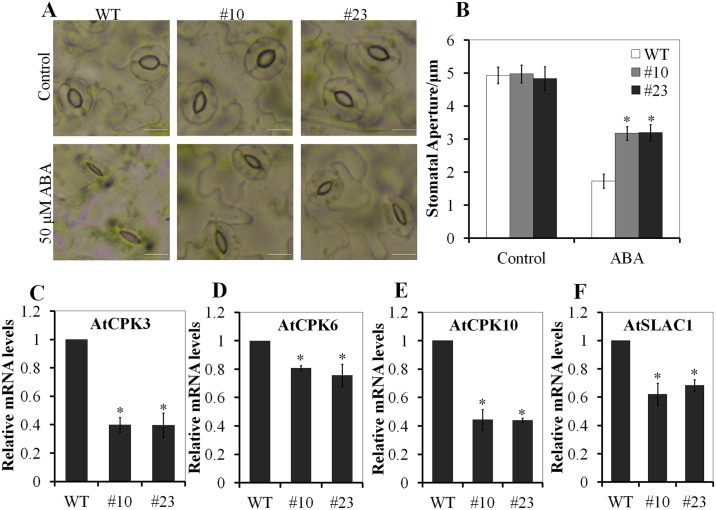
PnLRR-RLK27 reduced ABA-mediated stomatal movement in transgenic *Arabidopsis*. (A) Stomatal closure of the three lines after 50 μM ABA treatment for 2.5 h. (B) Statistical analysis of the three *lines* stomatal aperture shown in (A). (C, D, E and F) The expression levels of stomatal movement related genes in 2-week-old *Arabidopsis* seedlings after 50 μM ABA treatment for 2 h. *Bar* 10 μm.

Stomatal movement was mostly controlled by ABA, thereafter to improve plant adaptation to environmental stress [53]. The stomatal apertures were no obvious difference between 4 week-old transgenic *Arabidopsis* plants (#10 and #23) and the WT plants under the normal conditions. However, after 50 μM ABA treatment, the transgenic *Arabidopsis* plants (#10 and #23) exhibited significantly wider of stomatal aperture in comparison with the control plants (Fig 6A). The stomatal aperture were average 3.2 μm in the transgenic plants and about 1.7 μm in the WT plants after 50 μM ABA treatment (Fig 6B). Several genes such as *AtCPK3*, *AtCPK6*, *AtCPK10* and *AtSLAC1* play an important role in regulating stomatal movement [54–55]. The expression levels of these genes in the transgenic plants were also lower in comparison with the WT plants after ABA treatment (Fig 6C, 6D, 6E and 6F). These results confirmed that PnLRR-RLK27 decreased the sensitivity of transgenic plants to ABA.

### PnLRR-RLK27 increased antioxidant capacity in *Arabidopsis*

**Fig 7:**
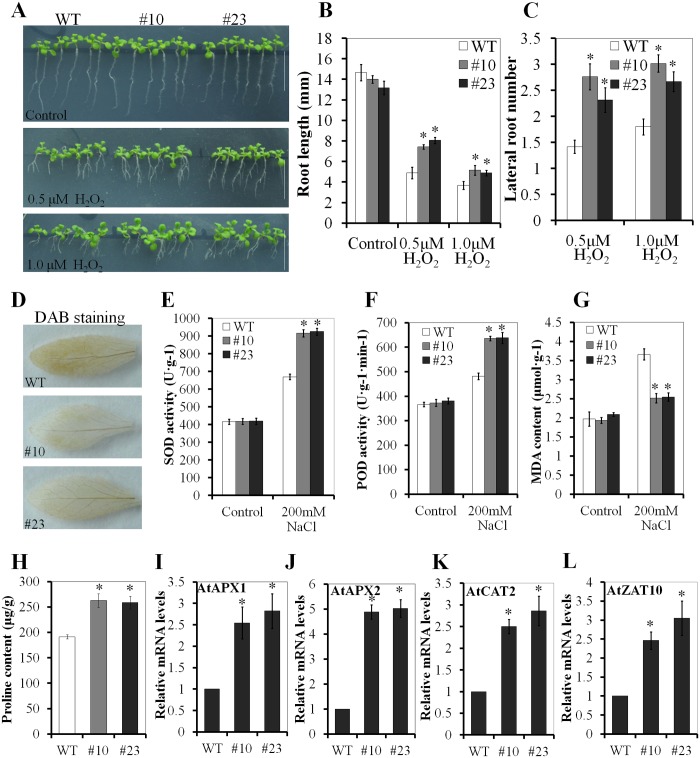
PnLRR-RLK27 enhances the oxidative tolerance and salt tolerance by promoting ROS-scavenging capacity and the expression levels of antioxidative-responsive genes in transgenic *Arabidopsis*. (A) The main root of transgenic lines were significantly longer than that of the control plants after 0.5 μM and 1.0 μM H_2_O_2_ treatment (10 days' seed germination). (B) Statistical analysis of the transgenic *Arabidopsis* seedling root length shown in (A). (C) Statistical analysis of the transgenic *Arabidopsis* seedling lateral root numbers shown in (A). (D) H_2_O_2_ levels of *Arabidopsis* by DAB staining in 4-week-old *Arabidopsis* plants after 200 mM NaCl treatment for 24 h. (E)and (F) POD and SOD activities in 4 week-old *Arabidopsis* plants after 200 mM NaCl treatment for 24 h. (G) the MDA levels in plants in 4-week-old *Arabidopsis* plants after 200 mM NaCl treatment for 24 h. (H) The content of proline in 2-week-old *Arabidopsis* seedlings after 200 mM NaCl treatment for 2 h. (I, J, K and L) The expression levels of antioxidative-responsive genes in 2-week-old *Arabidopsis* seedlings after 200 mM NaCl treatment for 2 h. Data are presented as means ±SE, and asterisks (*) indicate significant differences of means between the transgenic lines and the WT plants (*P*<0.05). *Bar* 10 mm.

Salinity stress usually causes the production of reactive oxygen species (ROS), and lead to oxidative stress. Therefore, tolerance to salinity stress has frequently been associated with tolerance to oxidative stress [20]. Results showed that the overexpression of *PnLRR-RLK27* in *Arabidopsis* improved resistance to H_2_O_2_ stress (Fig 7). The transgenic *Arabidopsis* plants (#10 and #23) exhibited longer primary roots in comparison with the WT plants in the presence of 0.5 and 1.0 μM H_2_O_2_ (Fig 7A). The root length of the transgenic plants was averagely 1.6-fold longer than that of the WT plants at 0.5 μM H_2_O_2_, while 1.4-fold longer at 0.75 μM H_2_O_2_ (Fig 7B). Meanwhile, the lateral root numbers in the transgenic *Arabidopsis* plants (#10 and #23) were averagely 1.7-fold more than the WT plants at 0.5 μM H_2_O_2_, while 1.6-fold more at 0.75 μM H_2_O_2_ (Fig 7C). The levels of ROS were also measured. After 200 mM NaCl treatment, the transgenic *Arabidopsis* plants (#10 and #23) exhibited lower H_2_O_2_ level, forming fewer brown H_2_O_2_ spots in leaves visualized by 3,3′-diaminobenzidine (DAB) staining (Fig 7D). PnLRR-RLK27 reduced the H_2_O_2_ levels possibly via activating scavengers. SOD and POD are such antioxidative enzymes to eliminate of H_2_O_2_. After 200 mM NaCl treatment, the activities of SOD and POD were significantly higher in the transgenic *Arabidopsis* plants (#10 and #23) than that in the WT plants (Fig 7E and 7F). Furthermore, the expression levels of ROS scavenging enzymes (such as AtAPX1, AtAPX2, AtCAT2 and AtZAT10) were significantly increased in transgenic *Arabidopsis* plants (#10 and #23) after salt treatment (Fig 7I, 7J, 7K and 7L). The content of malondialdehyde (MDA), an indicator of intracellular ROS damage, in the transgenic *Arabidopsis* plants (#10 and #23) was 33.8% less than in the WT plants (Fig 7G). The content of proline in the transgenic *Arabidopsis* plants (#10 and #23) was about 1.35-fold higher than that in the WT plants (Fig 7H).

### PnLRR-RLK27 increased the expression of stress related genes in transgenic *Arabidopsis* and *Physcomitrella patens*

**Fig 8:**
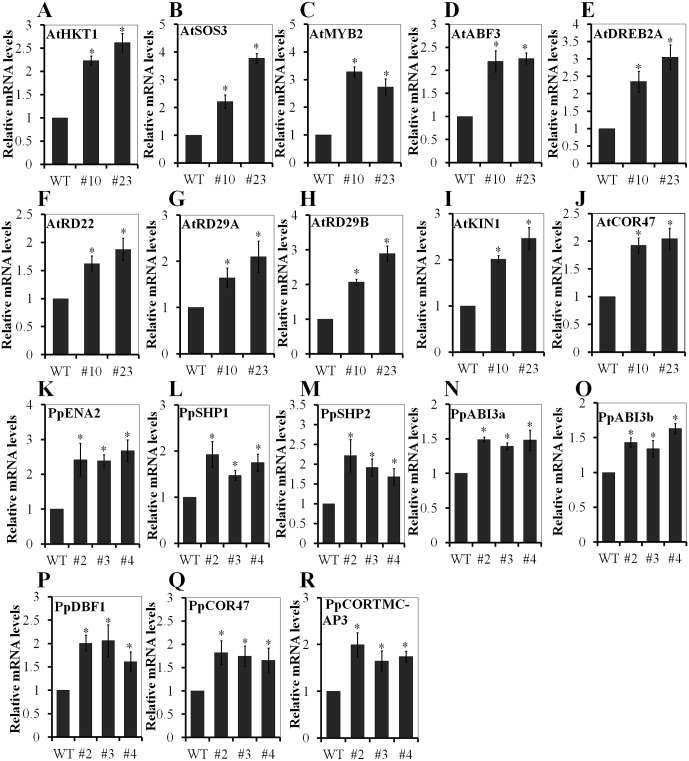
The stress-responsive genes expression pattern in PnLRR-RLK27 transgenic *Arabidopsis* and *Physcomitrella patens*. The expression levels of several abiotic stress/ABA-related genes were measured by qPCR analysis using the SYBR Green master mix (DBI). The *Arabidopsis* actin gene and *Physcomitrella patens* tubulin gene were served as normalization (S1 Table). Gene expression was analyzed using comparative Ct (2^-ΔΔCt^) method. The reactions were performed in triplicate.

To further elucidate the molecular mechanism of PnLRR-RLK27 increasing the salinity resistance in transgenic plants, the expression of several abiotic stress-related genes was measured by real-time PCR. After 2 h treatment with 200 mM NaCl, the transcription levels of salt tolerance genes *AtHKT1* and *AtSOS3*, stress-responsible genes *AtMYB2*, *AtABF3*, *AtDREB2A*, *AtRD22*, *AtRD29A*, *AtRD29B*, *AtKIN1* and *AtCOR47* in transgenic *Arabidopsis* were all significantly higher in transgenic plants (Fig 8). Meanwhile, the transcription levels of salt tolerance genes *PpENA2*, *PpSHP1* and *PpSHP2*, ABA-related genes *PpABI3a*, *PpABI3b*, and stress-responsible genes *PpDBF1*, *PpCOR47* and *PpCORTMC-AP3* in transgenic *P*. *patens* were also significantly increased in transgenic plants (Fig 8). Therefore, when plants were subjected to salt stress environment, PnLRR-RLK27 may enhance salt tolerance by upregulating several stress related genes.

## Discussion

Plant leucine-rich repeats receptor-like protein kinases (LRR-RLKs) play important roles in the signal perception, amplification and transduction to abiotic stress [2]. LRR-RLKs were mainly identified based on the whole-genome sequences of *Arabidopsis* and rice, but only a small part of them had been verified to possess clear functions [10, 56–59]. For example, the *Arabidopsis* LRR-II type RLK genes were induced by many environmental stresses, such as gravity, cold, high light, osmotic stress, auxin and abscisic acid, suggesting that they may participate in the general abiotic stress response [56]. The expression of rice LRR-RLKs (OsSIK1, OsGIRL1, OsLP2) were regulated by salt, drought, abscisic acid, salicylic acid, jasmonic acid or H_2_O_2_ stresses, indicating that rice LRR-RLKs might be involved in multiple signaling pathways regulating developmental and stress processes [4,20,22, 60]. In this study, through the tanscriptome sequencing, we identified 56 LRR-RLK genes from the Antarctic moss *P*. *nutans*. Of them, the expression of *PnLRR-RLK27* gene were induced by high salinity, cold, drought and ABA stresses, suggesting that it might be involved in the processes of the Antarctic moss *P*. *nutans* adopting to abiotic stresses (Fig 2). In *Arabidopsis* and rice, AtSERK and OsSERK family genes (the *Arabidopsis* and rice orthologs of PnLRR-RLK27) play important roles in plant somatic embryogenesis, development, BR signaling, stomatal patterning, plant immunity defense and senescence, but their functions in abiotic stresses were not well documented [61–64].

The Antarctica, with its almost pristine ecosystem and relatively simple vegetation, offers unique habitat for investigating the influence of environmental events on species performance [65]. Mosses are the dominant vegetation in ice-free coastal Antarctica. The importance of mosses for the research of the effects of abiotic stresses (such as cold, desiccation and UV-B radiation) on terrestrial plants has been well established and further propeled, since *Physcomitrella patens* genome was sequenced [66–67]. Recently, the advanced experimental tools support highly efficient and accurate gene targeting through homologous recombination in *P*. *patens* [68]. It makes us better understand the degree of evolution and conservation of plants. Furthermore, as a link between green algae with seed plants, the stress-associated signaling pathways in *P*. *patens* are functionally conserved, such as ABA signaling pathway, membrane proteins, molecular chaperones, redox-related functions and stomatal closure with the seed plants [68–70]. *P*. *patens* is highly resistance to drought, salinity and UV-B stresses, but their inherent molecular mechanism were mainly clarified at transcriptome levels [69,71]. Previously, a high-throughput Illumina high-throughput sequencing was used to analyze the gene expression profiles of *P*. *nutans* after cold treatment. Differential gene expression analysis indicated that 42-upregulated and 33-downregulated putative receptor-like kinases from *P*. *nutans* were response to cold stress [72]. Furthermore, we reported that a cytoplasmic-type RLKs (PnRLK-1) from the Antarctic moss *P*. *nutans* couldincrease salt and oxidative stress tolerance [32]. In addition, we also reported that a leucine-rich repeats RLKs (PnLRR-RLK) from the Antarctic moss *Pohlia nutans*, which belongs to LRR XI-type subfamily proteins, could improve salt and ABA stress tolerance [73]. In this study, through the overexpression in bryophyte *P*. *patens* and heterologous expression in *Arabidopsis*, we found that PnLRR-RLK27 might be as a signaling regulator enhancing plant tolerance to salt and oxidative stress associated with the positive regulation of the ABA-mediated signaling network.

Salinity levels adversely affect the growth and development of Antarctica mosses, as many plant species grow near the Antarctic coast. High salinity also causes both hypotonic and hyperosmotic stress and can lead to plant death, which has comprehensively restrained the crop production [74]. So, identifying novel genes and exploring their functions in stress adaptation are the basis for effective strategies to improve plants tolerance to salt stress. Interestingly, it is well known that the LRR-RLKs are one of important regulator for plant response to salt stress [19,22,58,75]. In this study, the expression of *PnLRR-RLK27* was induced by abiotic stresses (Fig 2). Transgenic phenotypic analysis showed that *PnLRR-RLK27* improved tolerance to salt stress with larger clone size in transgenic *P*. *patens*, higher seeds germination rates and longer primary roots in *Arabidopsis* (Fig 4). Salt resistance is highly correlated with their ability to reduce the accumulation of sodium ions in the shoot [76]. AtHKT1 is a salt tolerance determinant that controls Na^+^ entry and high affinity K^+^ uptake [49]. The *Arabidopsis* salt tolerance gene *SOS3* (for salt overly sensitive 3) encodes a calcium-binding protein function through the notable SOS pathway [76]. HKT1 and SOS3 both play a key role in the ionic transport [77]. In this study, the expression levels of *AtHKT1* and *AtSOS3* were significantly higher in transgenic plants (Fig 8). In addition, the *P*. *patens* sodium ATPase (PpENA2) are transmembrane transport proteins that mediate Na^+^ efflux and K^+^ influx into cells and play important role in maintaining cellular ion homeostasis in salt environments [78]. PpSHP1 and PpSHP2, AtRCI homology protein, are involved in the salt responses by avoiding over-accumulation of Na^+^ and K^+^ ions [79–80]. In this study, the expression levels of *PpENA2*, *PpSHP1* and *PpSHP2* were significantly higher in transgenic *P*. *patens* (Fig 8). Proline is a major organic osmolytes that can accumulate in plants in response to abiotic stresses (salinity, cold and drought) [81]. In this study, the contents of proline in transgenic *Arabidopsis* were higher than that in WT plants (Fig 7H). Thus, the up-regulated expression of these stress-responsive genes and accumulation of osmolytes in *PnLRR-RLK27* overexpression plants might be one of the reasons for the improvement of plants salt resistance.

Plants have evolved the ability to survive under high salinity by developing highly systematic signaling networks, in which ABA is one of the important regulators in improving plant tolerance to salt stress [82]. In this study, the transgenic *Arabidopsis* was highly insensitive to the provision of exogenous ABA (Fig 5D to 5G). In comparison with accumulated knowledge about ABA signaling pathway in higher plants, the ABA signaling components that response to stress tolerance were still not well characterized in bryophytes [83]. In this study, when gametophytes grew on BCD medium supplementted with 0.25 μM, 0.5 μM or 1 μM ABA, there were no obvious differences between the transgenic *P*. *patens* and the wild-type plants. However, the growth rates of three transgenic gametophytes were significantly higher than the WT plants on 10 μM or 15μM ABA medium (Fig 5A and 5B). Previously, 10, 50 or 100 μM ABA were also used to detect desiccation tolerance of *Physcomitrella patens* abi3 mutants [84–85], suggesting that mosses have a relatively higher resistance to ABA treatment. In addition, ABA negatively regulates the stomatal aperture by controling ion fluxes in guard cells. Typically, ABA activates kinases SnRK2s (OST1) and Ca^2+^-dependent protein kinase (CPK): CPK3/6/10, which phosphorylates the anion channel SLAC1, and finally leads to stomatal closing [54,86]. Moreover, the *Arabidopsis* SERKs could negatively regulate stomatal development by ligand-induced heteromerization and transphosphorylation with the ER and ERL1 receptors downstream of the EPF1 and EPF2 ligands and upstream of the YDA-MKK4/MKK5-MPK3/ MPK6 cascade [63]. The rice LP2 increased drought sensitivity by enhancing stomatal opening and stomatal density [22]. *Arabidopsis* GHR1 increased ABA- and H_2_O_2_- induction of stomatal closure by directly interacting with SLAC1, and GHR1, OST1 and CPKs coordinately regulated SLAC1 activity [39]. In this study, after ABA treatment, the stomatal aperture in transgenic *Arabidopsis* was significantly wider; the expression levels of *AtCPK3*, *AtCPK6*, *AtCPK10*, *AtSLAC1* in the transgenic plants were also lower in comparison with the WT plants (Fig 6). Thus, *PnLRR-RLK27* involved in regulating the ABA signaling pathway.

The generation of reactive oxygen species (ROS) is one of the most common plant responses to different stresses, representing a point at which various signaling pathways come together [87]. Its excess accumulation will result in oxidative damage of membrane lipids, DNA, proteins and carbohydrates, and causes toxic substance MDA production [88–89]. During evolution, plants cells have acquired different antioxidants and ROS-scavenging enzymes to cope with the increased levels of ROS [90]. ROS-scavenging mechanism of *P*. *patens* is less researched and its antioxidant system mainly includes chloroplast peroxidases and monodehydroascorbate reductase (MDHAR), cytochrome P450 monooxygenases, lipoxygenase (LOXs), 2-Cys peroxiredoxin and peroxiredoxin [91–93]. However, ROS-scavenging system of *Arabidopsis* is clear studied, and it has a complex and systematic ROS-scavenging mechanism [90]. ROS-scavenging system in *Arabidopsis* includes antioxidant molecules and antioxidant enzymes, such as superoxide dismutase (SOD), peroxidase (POD), catalase (CAT), and ascorbate peroxidase (APX) [94–95]. In this study, *PnLRR-RLK27* in transgenic *Arabidopsis* obviously enhanced the resistance to H_2_O_2_, and the levels of H_2_O_2_ were lower visualized by DAB staining (Fig 7D). The reactive aldehyde such as MDA is considered to reflect the degree of ROS-induced lipid peroxidation [96], thus the decreased MDA content in transgenic *Arabidopsis* suggested that *PnLRR-RLK27* could alleviate ROS-induced damage (Fig 7G). Plants have evolved a complex antioxidant system to detoxify ROS, in which ROS scavenging enzymes play essential roles in detoxifying stress-induced ROS [97–98]. SOD and POD are a group of these enzymes that catalyze the oxidation of many substrates at the expense of H_2_O_2_, finally lead to reduce of H_2_O_2_ [99–100]. AtAPX1, AtAPX2 and AtCAT2 are the antioxidant enzymes that can increase the plant resistance to oxidative stress [97–98]. AtZAT10 is a C2H2-zinc finger protein in *Arabidopsis* that can improve the expression of reactive oxygen-defense transcripts and enhance plants tolerance to salinity, heat and osmotic stress [101]. In this study, the activities of SOD and POD enzymes in transgenic *Arabidopsis* were higher than that in WT plants; the transcription levels of *AtAPX1*, *AtAPX2*, *AtZAT1* and *AtCAT1* were also significantly increased in transgenic *Arabidopsis* (Fig 7E, 7F, 7I, 7J, 7K and 7L). Therefore, these results suggested *PnLRR-RLK27* could activate ROS scavenger to protected cells from ROS-induced lipid peroxidation.

Many salinity-inducible genes have been reported, and most of them are regulated within ABA signaling pathway [102]. ABF3 is a master transcription factor that cooperatively regulate ABRE-dependent gene expression for ABA signaling under conditions of water stress [103]. DREB2A is a *trans*-acting activator of AP2/ERF transcription factor that can interact with AREB/ABF proteins to regulate the expression of stress- and ABA-inducible genes in *Arabidopsis* [104]. MYB2 is a *trans*-acting protein of R2R3-type MYB transcription factor that activates the dehydration- and ABA-inducible expression of the RD22 gene in *Arabidopsis* [105]. ABF3, DREB2A and MYB2 are upregulated by ABA, dehydration and high-salinity stresses [106]. The expression of several stress-induced genes (*RD22*, *RD29A*, *RD29B*, *COR47* and *KIN1*) was regulated by above transcription factors, and were activated when plants suffered by cold, drought, salt and ABA [107–108]. In this study, we found that the expression of *PnLRR-RLK27* markedly increased the transcripts of the major transcription factor (ABF3, DREAB2A and MYB2) and the stress-related genes (*AtRD22*, *AtRD29A*, *AtRD29B*, *AtKIN1* and *AtCOR47*) after salt treatment (Fig 8). In *P*. *patens*, PpDBF1, an AtDREB homolog, its expression enhanced higher tolerance to salt, drought and cold stresses [109]. PpCOR47 and PpCOR TMC-AP3 proteins are induced by drought, osmotic, salt and ABA stress in *P*. *patens* [68]. In this study, we found that the *PnLRR-RLK27* expression markedly increased the transcripts of the major transcription factor PpDBF1 and the stress-related genes (*PpCOR47* and *PpCORTMC-AP3*) after salt treatment (Fig 8). Recent studies also suggested that RLKs participate in ABA-related signal pathway and enhance stress tolerance [2,110]. For example, the *Glycine soja* GsCBRLK enhanced plant tolerance to ABA and high salinity by increasing the expression pattern of stress marker genes (*RD22*, *RD29A*, *KIN1*, *COR15A* and *NCED3*) in response to ABA and high salt [111]. In other family of genes, there is evidence that some genes confer salt stress tolerance and insensitivity to ABA. For example, OsbZIP71 (a bZIP transcription factor) overexpressing rice significantly improved salt, drought and PEG osmotic stresses tolerance and insensitivity to ABA with upregulating the expression of abiotic stress-related genes (*OsHKT6*, *COR413-TM* and *OsMyb4*) [112]. The abo3 (a WRKY transcription factor) mutant was less drought tolerance and hypersensitive to ABA in both seeds germination and seedling growth by downregulating the expression of *ABF2/AREB1*, *RD29A* and *COR47* [113]. Thus, our results suggested that *PnLRR-RLK27* also involved in plant tolerance to salinity stress by interacting with ABA-mediated signal and gene expression.

Since the first plant RLK gene (*ZmPK1* from maize) was cloned in 1993, the researchs on the ligand and downstream components of RLKs have been extensively concerned. Several LRR-RLKs (i.e., RLK7, BAK1, SOBIR1, GHR1 and RDK1) had been found to mediate plant immunity [114–116], stomatal movement [39] and ABA sensitivity [117] by phosphorylating downstream components (prePIP1 peptide, BIR2, RLP, SLAC1 or ABI1). Therefore, to further clarify the *PnLRR-RLK27* mediated stress signaling pathway in the *P*. *nutans*, identification of the extracellular ligand(s) and downstream components (ion channel, active polypeptide or transcription factor) were recommended. This will facilitate the improvements of abiotic stress tolerance in plant through genetic manipulation. In conclusion, our study provides a new insight that *LRR-RLK* participates in the process of *P*. *nutans* adapting and acclimating to the Antarctic adverse environments.

## Supporting information

S1 Table Primers for gene clone, plasmid construction and real-time PCR analysis. (DOC)

S2 Table PnLRR-RLKs transcriptional levels after salt treatment for 1 h. (XLSX)
