# Supplementary material for: PnLRR-RLK27, a novel leucine-rich repeats receptor-like protein kinase from the Antarctic moss Pohlia nutans, positively regulates salinity and oxidation-stress tolerance
Source: PLoS One. 2017 Feb 27;12(2):e0172869. doi: 10.1371/journal.pone.0172869 (PMC5328275; doi:10.1371/journal.pone.0172869)
Supplement: S1 Table — (DOC) [file pone.0172869.s001.doc]

Table S1 Primers for gene clone, plasmid construction and real-time PCR analysis.

| Primers | Sequence (5`-3`) | Annotation |
| --- | --- | --- |
| PnLRR-RLK27-qF | CAGCAAAGGGACTGGCATACT | Real-time PCR |
| PnLRR-RLK27-qR | CTCGAACTGCGGTCGTGATA |
| Pn-tubulin-qF | AGCGGTGTGACTTGTTGCTTACG | Real-time PCR |
| Pn-tubulin-qR | TCTGCTGGGTAAGTTCTGGGACG |
| PnLRR-RLK27 XbaI | GCTCTAGAATGATCATGCAGTCCAGGCTGCTTC | Construct pSTART |
| PnLRR-RLK27 KpnI | CGGGGTACCCTATCTTGGGCCAGAGAGCTCAATAG |
| PnLRR-RLK27 XhoI | CCGCTCGAGCCGTGACCGTCGTCAAGGATGATCATGC | Construct pBI221 |
| PnLRR-RLK27 KpnI | GGGGTACCTCTTGGGCCAGAGAGCTCAATAGCGTGCAG |
| PnLRR-RLK27 F  SmaI | CAGGCCGGCGCGCCACCCGGGATGATCATGCAGTCCAGGCTGCTTC | Construct  pTFH15.3 |
| PnLRR-RLK27 R SmaI | TCGGCCGGGCCCTCCCGGGCTATCTTGGGCCAGAGAGCTCAATAG |
| At-tubulin-F | TTTGTGCTCATCTTGCCACGGAAC | Semi-qPCR |
| At-tubulin-R | CTCAAGAGGTTCTCAGCAGTACC |
| AtHKT1-qF | TCAGTGCATATGGAAACGTTGG | Real-time PCR |
| AtHKT1-qR | CCATTGGACTCCATCGTCCTG |
| AtSOS3-qF | CGCTTCTTCACGAATCCGAACTAGTTC | Real-time PCR |
| AtSOS3-qR | GGCAAAGTCATGTTCTTGATGAGCGATG |
| AtMYB2-qF | AACGTCTTCGAATTCTCCGGCTGA | Real-time PCR |
| AtMYB2-qR | ATCGTTGAACTCTCCGAAACCCGT |
| AtABF3-qF | AACGCTGGGAGAGATGACTTTGGA | Real-time PCR |
| AtABF3-qR | TCCCAAGACCTCCATTACTGCCAA |
| AtDREB2A-qF | AAACCTGTCAGCAACAACAGCAGG | Real-time PCR |
| AtDREB2A-qR | TTAAGCCTGCAAACACATCGTCGC |
| AtRD22-qF | AGGAGCAAACCCTTTCGTGT | Real-time PCR |
| AtRD22-qR | CGTTTCAACGTCTCCGAAAA |
| AtRD29A-qF | CTTGATGGTCAACGGAAGGT | Real-time PCR |
| AtRD29A-qR | CAATCTCCGGTACTCCTCCA |
| AtRD29B-qF | AGAAGGAATGGTGGGGAAAG | Real-time PCR |
| AtRD29B-qR | CAACTCACTTCCACCGGAAT |
| AtKIN1-qF | CATCTCTTCTCATCATCACTAAC | Real-time PCR |
| AtKIN1-qR | AACATTGCTCTTCTCCTCAG |
| AtCOR47-qF | GGAAGAAGGAAGAGGAAGTG | Real-time PCR |
| AtCOR47-qR | GCTGTTGGATCGGTGAAG |
| PpENA2-qF | CCAACGCAATCAATGAAACACCT | Real-time PCR |
| PpENA2-qR | GTAATGACCGCGATAAGCCCCTC |
| PpSHP1-qF | CACGTTCGTTGATGTGCTGTTG | Real-time PCR |
| PpSHP1-qR | GCAAGTAACCCAGGATTGTCAG |
| PpSHP2-qF | TTAGCTATTCTGTTGCCGCCTCT | Real-time PCR |
| PpSHP2-qR | GACGTAAAGCGCATACAAGATCC |
| PpABI3a-qF | ATGCGTCGTTGTCTTCATATTTAAT | Real-time |
| PpABI3a-qR | GGGTTCATCTGAGGCGGGTTTA | PCR |
| PpABI3b-qF | AAAAGTCCCTGCCCATTCGTGAG | Real-time PCR |
| PpABI3b-qR | ACGTCTACAAAGGTGCCGCTACC |
| PpDBF1-qF | GAGGTCACGAATCTGGTTGGGCT | Real-time PCR |
| PpDBF1-qR | CGAGGAGGGCTGTCGGGGAAGTT |
| PpCOR47-qF | AAACGCCCGAGAGCGGTGTT | Real-time PCR |
| PpCOR47-qR | TGGTGGAGGCTGGAGCAGTGGA |
| PpCORTMC-AP3-qF | AACACCCCCCATGCCGACTTCT | Real-time PCR |
| PpCORTMC-AP3-qR | CTCCTTGCCCATACGCTGAACG |
| AtACTIN2/8-qF | GGTAACATTGTGCTCAGTGGTGG | Real-time PCR |
| AtACTIN2/8-qR | AACGACCTTAATCTTCATGCTGC |
| PpTUA1-qF | CGTAGGAGGGACCAGTTTGG | Real-time PCR |
| PpTUA1-qR | TGCATTCATCCCCGAGTCA |
| AtCPK3-qF | CTGAAGGTTATCGCTGAGA | Real-time PCR |
| AtCPK3-qR | AAGGTGACTATTCCATTGTTATC |
| AtCPK6-qF | CTGTTCATTCTCCTACTAC | Real-time PCR |
| AtCPK6-qR | GTCCTTGTCCTAACTTAC |
| AtCPK10-qF | CAGAGATTGTAGGAAGTC | Real-time PCR |
| AtCPK10-qR | CACAGAGCAAGATATAGATA |
| AtSLAC1-qF | ACAACAACAACAAGAGAT | Real-time PCR |
| AtSLAC1-qR | GATGATTCCACCTGATTC |
| AtAPX1-qF | GTCCATTCGGAACAATGAGGTTTGAC | Real-time PCR |
| AtAPX1-qR | GTGGGCACCAGATAAAGCGACAAT |
| AtAPX2-qF | TGATGTGAAGACGAAGACAGGAGGAC | Real-time PCR |
| AtAPX2-qR | CCCATCCGACCAAACACATCTCTTA |
| AtCAT2-qF | TCCCGTCGAGGTATGACCAGGTT | Real-time PCR |
| AtCAT2-qR | CTTGCCAGCTTCTGTCCCAAAGACT |
| AtZAT10-qF | TTTCCACCACCAAAACCTCACT | Real-time PCR |
| AtZAT10-qR | GTAGCTCAACTTCTCCACCGCC |
